# Prevalence of Bovine Leukemia Virus Antibodies in US Dairy Cattle

**DOI:** 10.1155/2018/5831278

**Published:** 2018-11-11

**Authors:** Rebecca M. LaDronka, Samantha Ainsworth, Melinda J. Wilkins, Bo Norby, Todd M. Byrem, Paul C. Bartlett

**Affiliations:** ^1^Department of Large Animal Clinical Sciences, College of Veterinary Medicine, Michigan State University, East Lansing, MI 48824, USA; ^2^North Star Cooperative, 4200 Forest Rd., Lansing, MI 48910, USA

## Abstract

**Objective:**

To estimate current US herd-level and animal-level prevalence of bovine leukemia virus (BLV) in dairy cows and characterize epidemiologic features.

**Design:**

Cross-sectional observational study design and survey.

**Animals:**

4120 dairy cows from 103 commercial dairy herds in 11 states across the US.

**Procedures:**

Milk samples were collected from dairy cows through routine commercial sampling and tested for anti-BLV antibodies by antibody capture ELISA. Based on the ELISA results of a sample of an average of 40 cows per herd, within-herd apparent prevalence (AP) was estimated by a directly standardized method and by a lactation-weighted method for each herd. Within-herd AP estimates were summarized to give estimates of US herd-level and animal-level AP. Differences in AP by lactation, region, state, breed, and herd size were examined to characterize basic epidemiologic features of BLV infection.

**Results:**

94.2% of herds had at least one BLV antibody positive cow detected. The average within-herd standardized AP was 46.5%. Lactation-specific AP increased with increasing lactation number, from 29.7% in first lactation cows to 58.9% in 4th and greater lactation cows. Significant differences were not observed based on region, state, breed, or herd size.

**Conclusions and Clinical Relevance:**

These results are consistent with a historical trend of increasing prevalence of BLV among US dairy cattle. Given the findings of other studies on the negative impacts of BLV infection on milk production and cow longevity, these findings are clinically relevant for veterinarians counseling dairy clients on the risks of BLV to their herds.

## 1. Introduction

Bovine leukemia virus was recognized as the causative agent of enzootic bovine leukosis in the late 1960s and early 1970s [1–4]. In the US, where there is no control program for BLV, the animal-level prevalence in the dairy cattle population has increased steadily from approximately 10% at that time to over 40% today [5–12]. Other major dairy producing countries that have not implemented large-scale eradication programs, including Canada, Argentina, Japan, and China, have also reported BLV prevalence in their dairy herds of 30-50% [11, 13–21]. In contrast, 19 member states of the European Union, the United Kingdom, New Zealand, and Australia have completely eradicated BLV from their dairy cow populations, and control programs are underway in the remaining member states of the EU resulting in an overall herd prevalence in the EU of less than one percent [11, 22, 23].

While some previous studies do not show significant negative effects of BLV infection on production and longevity or survival [13, 24, 25], the majority of recent studies have reported a negative association between BLV infection and both milk production and cow longevity [21, 22, 26–32]. Between 2010 and 2012, our research group conducted studies of BLV in 113 Michigan dairy herds using milk ELISA1 testing of a sample of 40 cows in each herd to estimate within-herd AP, which ranged from 0% to 80.6% with an average of 32.8% [9, 33–36]. The cows were then followed for an average of 597 days to evaluate longevity and milk production. ELISA-positive cows in that study were 23% more likely to be culled during the follow-up period, and 2nd and greater lactation ELISA-positive cows had a projected 305-day mature equivalent milk production that was 177.3 kg less than their ELISA-negative herdmates [34, 36].

It therefore appears that BLV may be eroding the profitability and long-term sustainability of US dairy farms in ways that were unappreciated in previous decades when the US and many other nations opted not to control BLV [7, 22, 37–40]. It has been over a decade since the last national estimate of US BLV prevalence was made [8, 12]. An updated estimate of BLV prevalence in the US dairy industry is needed to fully understand the impact of these production effects on the industry today. The purpose of this study was to estimate the current US herd-level and animal-level BLV prevalence in our nation's dairy population and to characterize the basic epidemiologic features of infection.

## 2. Methods

This study used a cross-sectional study design in which herds were enrolled through their DHI membership. The protocol was approved by the Michigan State University Animal Care and Use Committee. Partnerships were established with DHI organizations and/or university extension agents in Wisconsin, Idaho, New York, Pennsylvania, Texas, Minnesota, Michigan, Ohio, Vermont, Utah, and North Carolina.

In each state, herds were enrolled in each of 3 herd-size categories: 70 to 199, 200 to 999, and greater than 1000 cows per herd. The proportion of herds in each category was established based on the proportions of cows in the state in herds of each size, as obtained from National Agriculture Statistics Service 2012 Agriculture Census data [41]. Additional inclusion criteria specified that herds should have a minimum of 70 milking cows, either be enrolled in DHI testing with at least 10 milk weights per year or have a milking system that records milk weights for individual cows, and have herd records available on one of four software programs^2^^,^^3^^,^^4^^,^^5^. The minimum herd size was established as an attempt to ensure that there would be at least 10 cows in milk in each of the 1st, 2nd, 3rd, and ≥4th lactation groups. The DHI staff or extension agents selected a convenience sample of herds in their service areas that fit the inclusion criteria and the herd size goals for the state. A letter of invitation was sent to the designated contact person for the herd, along with an informed consent form authorizing collection of samples and longitudinal collection of production data and a mailed survey of management practices adapted from an in-person interview form which was used in a previous study of risk factors for herd BLV prevalence [35]. DHI staff assisted in reminding herd contact people to complete these forms in a timely manner and replacement forms were supplied as necessary.

Within each herd, 10 cows in the 1st, 2nd, 3rd, and ≥4th lactations were targeted for sample collection, for a total of 40 cows per herd. The 10 targeted cows in each lactation group were those that were most recently calved, with the exception that sampled cows must have been ≥10 days after calving. As such, the sample of 40 cows was completely prescribed with no opportunity for the producer to selectively choose which cows would be sampled. If a milk sample for a targeted cow was not available for testing, an appropriate substitute was selected by DHI and research staff. In some cases, there were more or less than 10 cows sampled in each lactation group, because of availability of targeted cows and inclusion of substitutes. This sampling scheme followed the methods established in a study of 113 dairy herds in Michigan [9]. Milk samples from the selected cows were collected via routine DHI milk sampling protocols. Samples were collected in individual vials with preservative (bronopol/natamycin) and shipped to their respective local DHI labs for routine milk component analysis. Following component analysis, the remaining milk sample was shipped from the local DHI labs to the local lab of our research partner6 for BLV analysis using a commercially available antibody capture ELISA^1^ routinely used for serum and milk analysis [9, 33–36]. Samples were shipped and stored at ambient temperature and ELISA testing was completed within 18 days from the date of sample collection. Briefly, antibodies to BLV in diluted milk samples (1:30 sample buffer diluted to reduce the effect of carryover contamination) were captured with an ultrapure virus lysate and detected by reaction with horseradish-peroxidase-labeled monoclonal antibodies to bovine immunoglobulin after washing. Bound antibodies were detected by addition of enzyme substrate. Reaction times were standardized to the color development of positive controls (0.9 < 450nm OD < 1.2) and stopped by the addition of 0.5 N H_2_SO_4_. Samples with corrected OD (raw sample OD minus negative control OD) of ≥ 0.1 were considered positive. Using this 0.1 cutoff, this assay has nearly perfect agreement (*Κ* = 0.86) with the serum ELISA which has sensitivity and specificity of 99.8% and 100%, respectively, using AGID as the reference test [42, 43].

Herd-level AP was calculated as the proportion of herds with at least one ELISA-positive animal in the 40-cow sample. Within each herd, lactation-specific APs were calculated as the proportion of ELISA-positive cows in each lactation group. A directly standardized estimate of within-herd BLV AP (standardized AP) was calculated for each herd based on a theoretical standard population with 25% of the animals in each of the 1st, 2nd, 3rd, and ≥4th lactation groups, which is equivalent to the simple arithmetic mean of the lactation-specific APs. The standardized AP was validated as part of the BLV Herd Profile developed in our previous study of 113 Michigan dairy herds [9]. A lactation-weighted estimate of within-herd BLV AP (lactation-weighted AP) was also calculated for each herd as the average of lactation-specific APs weighted by the proportion of the herd in each lactation group. The standardized AP and lactation-weighted AP were calculated using a spreadsheet7; all other data analyses were conducted using statistical software8. Herd and cow characteristics and survey responses were summarized using PROC UNIVARIATE9 [44] and PROC FREQ10 [44]. Associations between standardized AP and herd state, region, and size were examined using PROC GLM for unbalanced ANOVA; where the overall ANOVA was significant, pairwise comparisons were examined using the Tukey-Kramer adjustment for multiple comparisons^11^ [44]. The models for herd state and region met the assumptions of equal variance and normality of residuals. The model for herd size had unequal variance among the herd size categories and therefore Welch's ANOVA^11^ [44] was used to test differences in group means; residuals were normally distributed. The proportions of ELISA test-positive and negative results by breed were examined using Fisher's exact test^10^ [44]. P values of < 0.05 were considered statistically significant.

## 3. Results

A total of 103 herds were enrolled in 11 states, 37 herds in the East (New York, Pennsylvania, Vermont, and North Carolina), 59 herds in the Midwest (Minnesota, Wisconsin, Michigan, and Ohio), and 7 herds in the West (Texas, Utah, and Idaho). Characteristics of enrolled herds are summarized in Table 1. Herd size ranged from 59 cows to 7453 cows; for the one herd that was below the established minimum of 70 cows, DHI staff confirmed that 10 cows in each lactation group were available for sampling prior to enrollment. Herds in the West tended to be larger than those in the East and Midwest. The majority of herds were predominantly Holstein, though several herds had large proportions of non-Holstein cows. The survey response rate among enrolled herds was 82.5%. From the survey results (Table 2), 65% of the herds reported being closed herds, the majority (78%) milked in a parlor or rotary milking facility, and just over half (52%) of herds reported using any pasture with the median length of pasture use in those herds being 6 months. Only 13% of herds reported testing at least one animal per year for BLV and 89% perceived BLV to be either not a problem or an insignificant problem within the herd.

A total of 4,120 cows, an average of 40 cows (range: 25-48) in each of 103 herds, were tested for BLV antibodies by milk ELISA. Due to the protocol of sampling the 10 most recently calved cows in each lactation group, the distribution of days in milk was skewed to the right (mean: 59 days; median: 33 days), especially in smaller herds and in the 3rd and ≥4th lactation groups. Holstein cows made up 94% of the tested animals; the remainder of the cows were crossbred (4%), Jersey (2%), and less than one percent of other breeds including Brown Swiss and Guernsey. The proportion of ELISA-positive animals did not significantly differ by breed (Fisher's exact test, p=0.141).

**Figure 1:**
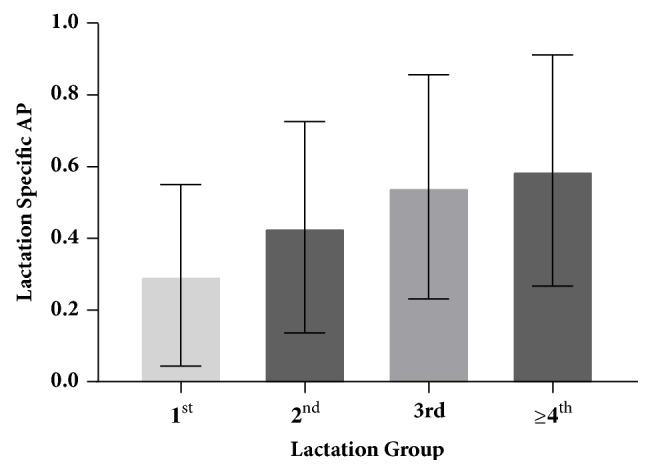
Mean lactation-specific APs with standard deviation bars by lactation group.

**Figure 2:**
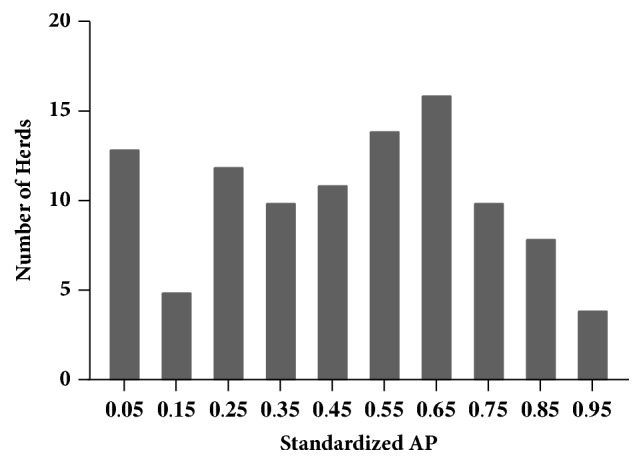
Frequency distribution of standardized AP; mean, 0.465; standard deviation, 0.27; histogram bin width=0.1; labels are at center point of bins.

The herd-level AP was 94.2%; i.e., 97 of 103 herds had at least one BLV ELISA-positive animal detected. Six herds had no ELISA-positive cows among those sampled and therefore all estimates of AP in those herds were 0%. The mean lactation-specific APs for the 1st, 2nd, 3rd, and ≥4th lactation groups were 29.7%, 43.1%, 54.4%, and 58.9%, respectively (Figure 1). For within-herd AP estimates, the standardized AP ranged from 0 to 96.9% with a mean of 46.5% (Figure 2); the lactation-weighted AP ranged from 0 to 98.5% with a mean of 42.4%. The standardized AP was on average 4.1% higher (range: -4.7% to 17.7%) than the lactation-weighted AP, depending on the age distribution and lactation-specific APs of the herd.

**Figure 3:**
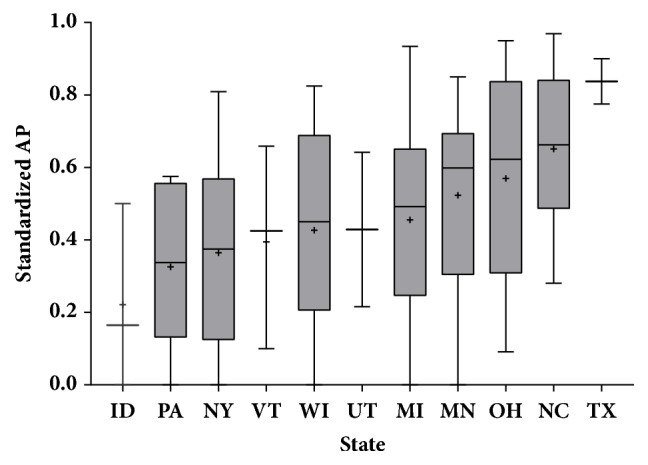
Distribution of standardized AP by state; significant ANOVA (p=0.025); no significant pairwise comparisons. For boxplots: + symbol=mean, midline=median, shaded box=interquartile range (where n>3), whiskers=min and max.

The distribution of standardized APs by state is shown in Figure 3. There were significant differences among states overall (ANOVA, p=0.025); however, none of the individual pairwise comparisons were significant using a Tukey-Kramer adjustment for multiple comparisons. When grouped into regions, there was no difference among Western, Midwestern, and Eastern herds (ANOVA, p=0.647). There was no significant association between standardized prevalence and herd size with herd size as a categorical variable (Welsh's ANOVA, p=0.881). However, the range of standardized prevalence was greater within the small herd size category (less than 200 cows). Five of the 6 herds where BLV was not detected were less than 200 cows, as was the herd with the highest prevalence estimate.

## 4. Discussion

In the late 1960s and early 1970s it was discovered that enzootic bovine leukosis, a disease recognized to cause lymphoid tumor development for over a century [45, 46], was caused by a novel virus [1–4]. Shortly after the time of its discovery, Baumgartener et al. [5] estimated the prevalence of antibodies to BLV in the US cattle population using AGID. In the study of 4,394 dairy cows in 100 herds in 5 states, 66.0% of herds were found to have at least one cow that was antibody-positive for BLV and 10.2% of cows were antibody-positive [5]. Since this initial estimate, studies of BLV AP on a national level in the United States have been rare. The 1996 NAHMS Dairy Study found that 88.3% of dairy herds had at least one antibody-positive animal, and 40.8% of cows overall were antibody-positive for BLV [7, 8, 12]. The 2007 NAHMS Dairy Study detected anti-BLV antibodies in bulk tank milk samples from 83.9% of participating dairy herds, but did not collect individual cow samples for an estimate of cow-level antibody prevalence [8]. The national study presented here, with a herd-level AP of 94.2% and mean standardized AP of 46.5%, is consistent with a continuation of the historical trend of a persistent proliferation of BLV US dairy herds.

A major advantage of the current study is that herds have been enrolled from 11 states in different areas of the country, placing it amongst the few studies that have examined BLV prevalence in US dairy cattle on a national level. These 11 states combined accounted for 51.1% of total milk production and 72.1% of licensed dairy herds in the US in 2011 [47]. The lack of Californian herds, which produce greater than 20% of the total milk in the country [47], is a potential weakness of the study. Even so, this study presents results from a broad context of many states and geographical regions and is the only estimate of BLV prevalence in the US dairy industry in the last decade. Like most of the other studies of BLV prevalence, the volunteer nature of enrollment may limit the scope of inference. It is possible that dairy producers who chose to enroll their herds in this study had a particular interest in BLV, leading to a biased estimate of prevalence. However, based both on previous studies and on the survey data from the current study, this seems unlikely. Firstly, the results of this study are consistent with the trends and results found in the work of our predecessors and contemporaries and therefore are likely not highly biased in either direction. Additionally, the survey responses of the dairy producers in this study reflected a low level of awareness and concern about BLV as a problem in their herds (Table 2), indicating that they generally had no particular interest in BLV.

The findings of this study were reported in terms of AP, meaning the prevalence of BLV antibody ELISA test-positive animals. For diseases which have a period of infection, immune response, and then recovery, the presence of antibodies simply indicates previous exposure to a pathogen and is not an appropriate test for prevalent infection. However, BLV produces lifelong persistent infection; therefore, the presence of antibodies is an appropriate test for determining disease prevalence [22]. As no diagnostic test for BLV is 100% sensitive and specific, some false-negatives and false-positives are assumed; therefore, AP is not perfectly equivalent to the true disease prevalence. Other large studies of BLV, including the NAHMS and Baumgartener studies and our previous work in Michigan [5, 8, 9, 12], have used prevalence of ELISA or AGID test-positive animals as estimates of disease prevalence. In this study, as in most production settings, whole herd BLV tests were cost prohibitive; therefore within-herd AP was estimated. In previous work by our group in which the protocol was established, it was calculated that with a sample size of 28 cows there was a 95% probability of detecting at least one positive cow in a herd with at least 10% prevalence, and the sample size was then increased to 40 cows to improve precision [9]. As methods of within-herd AP estimation, the standardized AP and lactation-weighted AP have both advantages and limitations. The standardized AP is directly comparable among herds, as well as among state, region, and herd size groups and within the same herd over time. Since cows in later lactations are more likely to be BLV positive, the lactation distribution of cows in the herds needs to be accounted for to avoid confounding. Just as age-adjusted death rates allow comparisons of mortality rates in populations with different age distributions by applying age-specific death rates to a standard population, the standardized AP allows comparisons of BLV prevalence among dairy cattle populations by applying lactation-specific APs to a standard herd population [48]. A second advantage of the standardized AP is that the calculation required for applying lactation-specific APs to the theoretical standard population is equivalent to taking a simple mathematical average of the four lactation-specific APs and is therefore a simple calculation that does not require knowledge of the herd's age structure or specialized knowledge to be utilized in a field setting.

The main limitation of the standardized AP is a direct consequence of its main advantage. Cows in later lactations make up the same proportion of the theoretical standard population as do cows in earlier lactations, even though they typically make up a smaller proportion of the actual population of most herds. Because cows in later lactations are also more likely to be BLV positive, the standardized AP tends to overestimate the true AP that could be obtained via a whole herd test. Therefore, we also calculated the lactation-weighted AP, where the proportion of the herd in each lactation group was used as the weight for that group in taking a weighted average of the lactation-specific APs. The lactation-weighted AP provides a more accurate estimate of the true AP given a herd's lactation distribution at a given point in time. As such, this estimate is more appropriate than the standardized AP for applications where the prevalence estimate is being used to predict some outcome, for example, the economic impact of BLV in a herd. The standardized AP was the primary estimate of BLV AP presented in this work because comparisons were made among states and by herd size, as well as between this study's findings and those of previous studies. In the latter case, caution must be used because, except our group's previous work in Michigan dairies which also utilized the standardized AP method [9], methods of calculating or estimating prevalence may not be consistent from one study to the next.

Examining prevalence of different subgroups of herds and cows can give an indication of factors that predispose to higher or lower BLV prevalence. In this study, prevalence was compared by lactation, breed, region, state, and herd size. Previous studies have also examined prevalence in these subgroups; however, in studies not using standardized AP as the measure of BLV prevalence, any comparisons between herds may have been confounded by the age distribution of cows in the herd. The lactation-specific APs in this study increased in the higher lactation groups, indicating that older cows were more likely to be BLV ELISA-positive. This is consistent with all other studies of BLV prevalence and is easily explained, recognizing that BLV infection is life-long and older cows have had more opportunity for exposure. In this study there were no differences in the proportion of ELISA-positive animals observed by breed, and there was no difference in standardized AP by herd region. There was also no difference in standardized AP by herd size; however it is notable that 5 of the 6 herds where BLV was not detected were smaller herds. The association between herd size and herd prevalence is less easily explained and not consistent across all studies. Baumgartener et al. found that smaller herds tended to have higher within-herd prevalence, while our prior study of Michigan dairy herds and both the 1996 and 2007 NAHMS studies found that larger herds tended to have higher prevalence [5, 8, 9, 12]. It seems unlikely that herd size itself would be a risk factor for higher within-herd BLV prevalence, but rather that the risk association is due to intermediate factors, such as herd management practices, which may be more or less common in herds of different sizes in specific study populations. Further study would be needed to determine what these intermediate factors may be. Possible explanations for the finding that herds with no detected BLV tended to be small in this study may include an increased likelihood that if a small herd has been closed for generations, it may never have introduced BLV into the herd or that in a small closed herd with low prevalence, at some point the last infected animal may have been culled by chance, inadvertently eradicating the infection from the herd. Examining how some herds have been able to maintain low or no BLV prevalence would be a direction for future research.

Results on differences among states in this study were conflicting in that the overall ANOVA was significant indicating that there are in fact real differences in standardized AP among herds by state, whereas there were no significant pairwise comparisons between any two states leaving us unable to say that any particular state has higher average standardized AP than any other. The small number of herds in some states (only 7 herds total in Texas, Utah, and Idaho combined) may have resulted in insufficient power to detect significant pairwise comparisons. The NAHMS 1996 Dairy Study found that the Southeastern region (comprising Kentucky, Tennessee and Florida) had higher BLV seroprevalence than other regions in the study [12]. While not statistically significant, the results of this study are consistent with that finding in that the 3 states in this study nearest the Southeast (Ohio, North Carolina, and Texas) had the 3 highest average standardized APs. One possible explanation for this observation is that BLV can be spread by biting flies. Southeastern states may tend to have longer fly seasons or heavier fly burdens, which may lead to increased BLV transmission among herdmates, resulting in higher within-herd prevalence [35, 49–51]. Further study is necessary to evaluate that hypothesis and to examine finer scale climatic factors associated with vector population dynamics.

## 5. Conclusion

The objectives of the current study were to document the prevalence of BLV in a national sample of dairy herds and to provide a basic epidemiologic description of the infection in the US dairy industry. The mean standardized AP of BLV reported here, 46.5%, is higher than previously reported in the US by any other large, multiregion study and is consistent with an ongoing trend of increasing prevalence over the last 5 decades. This finding, together with all the current evidence from the US and other countries with no established BLV control program, points to the prospect of ever increasing BLV prevalence. As the prevalence of BLV increases, the cumulative economic loss associated with infection will also continue to increase, making it a threat to the long-term sustainability of the US dairy industry. Future BLV research should therefore focus on controlling BLV transmission and reducing BLV prevalence.

## Figures and Tables

**Table 1:** Characteristics of enrolled herds from herd records; s.d.: standard deviation; i.q.r.: interquartile range.

		**East**	**Midwest**	**West**	**Total**
**Herd count (n)**		37	59	7	103

**Herd Size (cows per herd)**	**Mean (s.d.)**	509.7 (509.1)	558.9 (1020.5)	651.3 (496.0)	547.5 (836.3)
	**Median (i.q.r.)**	221.0 (560.0)	241.0 (586.0)	477.0 (151.0)	267.0 (560.0)
	**Range**	82 – 1832	59 - 7453	339 - 1764	59 -7453

**Proportion Holstein**	**Mean (s.d.)**	0.90 (0.20)	0.96 (0.10)	0.96 (0.50)	0.93 (0.14)
	**Median (i.q.r.)**	0.98 (0.10)	0.99 (0.03)	0.99 (0.08)	0.99 (0.04)
	**Range**	0.06 – 1.00	0.37 – 1.00	0.89 -1.00	0.06 -1.00

**Table 2:** Characteristics of enrolled herds from survey results; s.d.: standard deviation; i.q.r.: interquartile range.

**Survey response rate **%** (n)**		82.5 (85)

**Closed herd (No heifers, cows, or bulls added to the herd in the last 12 months) **%		64.7

**Type of milking facilities **%	**Parlor or Rotary**	77.7
	**Stanchion or Tie-stall**	18.8
	**Robotic **	2.4
	**Other**	1.2

**Pasture ever used **%		51.8

**For herds using pasture (n=43): Number of months used**	**Mean (s.d.)**	7.7 (2.8)
	**Median (i.q.r.)**	6 (6.0)
	**Range**	4 – 12

**Testing for BLV (At least 1 animal/year) **%		12.9

**Perception of BLV as a problem within the herd **%** (n=80)**	**“It is not a problem”**	47.5
	**“It is a problem, but not significant”**	41.3
	**“It is a significant problem, but not one of the biggest problems in this herd”**	10.0
	**“It is one of the biggest problems in the herd”**	1.3

## Data Availability

The ELISA test result, survey responses, and herd production record data used to support the findings of this study have not been made available to protect the confidentiality of participating dairy producers. Anonymized or aggregate data may be available from the corresponding author upon request.

## Endnotes

1. IDEXX Laboratories, Westbrook, ME.

2. PC Dart, Dairy Records Management Systems, Raleigh, NC.

3. Dairy Comp 305, Valley Agricultural Software, Tulare, CA.

4. DHI-Plus, DHI Computing Service, Provo, UT.

5. Accu-Trac Dairy Herd Manager, Micro Technologies, Amarillo, TX.

6. NorthStar Michigan Lab, NorthStar Cooperative, Grand Ledge, MI.

7. Microsoft Excel 2016, Microsoft Corp., Redmond, WA.

8. SAS, version 9.4, SAS Institute Inc, Cary, NC.

9. PROC UNIVARIATE, SAS, version 9.4, SAS Institute Inc, Cary, NC.

10. PROC FREQ, SAS, version 9.4, SAS Institute Inc, Cary, NC.

11. PROC GLM, SAS, version 9.4, SAS Institute Inc, Cary, NC.

## Abbreviations

AGID: Agar gel immunodiffusion
ANOVA: Analysis of variance
AP: Apparent prevalence
BLV: Bovine leukemia virus
DHI: Dairy herd improvement
ELISA: Enzyme-linked immunosorbent assay
NAHMS: National Animal Health Monitoring System
OD: Optical density.
